# Study protocol: cluster randomized trial of consultation strategies for the sustainment of mental health interventions in under-resourced urban schools: rationale, design, and methods

**DOI:** 10.1186/s40359-022-00733-8

**Published:** 2022-02-07

**Authors:** Ricardo Eiraldi, Barry L. McCurdy, Muniya S. Khanna, Courtney Benjamin Wolk, Henry A. Glick, Quinn A. Rabenau-McDonnell, Rachel Comly, Laura E. Rutherford, Jayme Banks, Steven A. Rufe, Kristina M. Popkin, Tara Wilson, Kathryn Henson, Abraham Wandersman, Abbas F. Jawad

**Affiliations:** 1 Children’s Hospital of Philadelphia, Roberts Center for Pediatric Research, 2716 South Street, Room 8293, Philadelphia, PA 19146-2305 USA; 2 Department of Pediatrics, University of Pennsylvania Perelman School of Medicine, 3400 Civic Center Boulevard, Philadelphia, PA 19104 USA; 3 Philadelphia College of Osteopathic Medicine, 4170 City Avenue, Philadelphia, PA 19131 USA; 4 OCD and Anxiety Institute, 3138 Butler Pike # 200, Plymouth Meeting, PA 19462 USA; 5 Department of Psychiatry, University of Pennsylvania Perelman School of Medicine, 3535 Market St., Philadelphia, PA 19104 USA; 6 Leonard Davis Institute of Health Economics, University of Pennsylvania, 3641 Locus Walk # 210, Philadelphia, PA 19104 USA; 7 Wharton School, University of Pennsylvania, 3620 Locust Walk, Philadelphia, PA 19104 USA; 8 Devereux Center for Effective Schools, 2012 Renaissance, Blvd., King of Prussia, PA 19406 USA; 9 School District of Philadelphia, 440 North Broad Street, Philadelphia, PA 19130 USA; 10 Department of Psychology, University of South Carolina-Columbia, Pendleton Street, Barnwell College, Suite #220, Columbia, SC 29208 USA

**Keywords:** Sustainment, Implementation, PBIS, Mental health supports, Urban schools, Effectiveness

## Abstract

**Background:**

The school is a key setting for the provision of mental health services to children, particularly those underserved through traditional service delivery systems. School-wide Positive Behavioral Interventions and Supports (PBIS) is a tiered approach to service delivery based on the public health model that schools use to implement universal (Tier 1) supports to improve school climate and safety. As our prior research has demonstrated, PBIS is a useful vehicle for implementing mental and behavioral health evidence-based practices (EBPs) at Tier 2 for children with, or at risk for, mental health disorders. Very little research has been conducted regarding the use of mental health EBPs at Tier 2 or how to sustain implementation in schools.

**Methods/design:**

The main aim of the study is to compare fidelity, penetration, cost-effectiveness, and student outcomes of Tier 2 mental health interventions across 2 sustainment approaches for school implementers in 12 K-8 schools. The study uses a 2-arm, cluster randomized controlled trial design. The two arms are: (a) Preparing for Sustainment (PS)—a consultation strategy implemented by school district coaches who receive support from external consultants, and (b) Sustainment as Usual (SAU)—a consultation strategy implemented by school district coaches alone. Participants will be 60 implementers and 360 students at risk for externalizing and anxiety disorders. The interventions implemented by school personnel are: Coping Power Program (CPP) for externalizing disorders, CBT for Anxiety Treatment in Schools (CATS) for anxiety disorders, and Check-in/Check-out (CICO) for externalizing and internalizing disorders. The Interactive Systems Framework (ISF) for Dissemination and Implementation guides the training and support procedures for implementers.

**Discussion:**

We expect that this study will result in a feasible, effective, and cost-effective strategy for sustaining mental health EBPs that is embedded within a multi-tiered system of support. Results from this study conducted in a large urban school district would likely generalize to other large, urban districts and have an impact on population-level child mental health.

*Trial registration* ClinicalTrials.gov identifier number NCT04869657. Registered May 3, 2021.

## Background

A major goal of dissemination and implementation research is to identify implementation strategies that can help to sustain the gains that occur as a result of the initial implementation of evidence-based practices (EBPs) [1]. The school is a key setting for the delivery of mental health services to children, particularly those who are underserved [2]. School-wide positive behavioral interventions and supports (PBIS) [3] is a service delivery framework based on the public health model used in schools to improve school climate and safety. This framework is a useful vehicle for implementing targeted (Tier 2) mental health EBPs for children with or at risk for mental health disorders [4–8]. PBIS is effective in reducing office discipline referrals (ODR) and improving children’s behavior [9] and overall school climate [10]. Very little research has been conducted on using mental health EBPs at Tier 2 or how to sustain their implementation [11]. The main aim of the study is to compare implementation, cost-effectiveness, and student outcomes of Tier 2 mental health interventions across 2 sustainment approaches for school implementers in 12 K-8 schools.

## Sustainment of EBPs in schools

EBPs can be sustained in under-served settings when there is internal capacity and ongoing opportunities for training. *Sustainment* has been defined as the maintenance of health benefits over time [12] and as the continued implementation of an intervention or prevention program that maintains fidelity to the core program principles after the supplemental resources that supported initial training are removed [13]. Studies examining implementation of PBIS have noted that adequate and ongoing training opportunities are frequently identified as “essential practices” needed to successfully sustain interventions [14, 15]. A lack or significant reduction of training resources has been associated with practice abandonment in the school setting [16]. Also, urban schools are typically unable to sustain mental health programs unless they are provided ongoing expert support [17]. Continuous training leads to the development of school-level expertise that can mitigate the negative effects of staff turnover [18]. Such training may increase participants’ abilities to implement interventions with fidelity and adapt the practice as needed without eliminating key components [13]. Preparing for sustainment concomitantly with initial implementation [19] with a goal of infusing the intervention into the school organizational culture is key [1]. Analyses of interventions in both mental health and school settings suggest that sites are more likely to sustain interventions if they are implemented with high rates of fidelity [20], and if implementation and student-outcome data are used to inform decision-making [20, 21]. Sustainment is more likely when EBPs are supported at the district level and resources are dedicated to developing a model for implementation [22]. Reviews of the literature have consistently identified the importance of a program “champion” in intervention sustainment [23]. Also, school principals have been identified as critical to intervention sustainment [24] and can provide important support as both instructional and school-culture leaders.

### Proposed sustainment strategies

Little is known about what models of support for sustainment are effective, feasible, and cost-effective. In order to achieve the potential public mental health benefits of targeted mental health interventions in schools, an efficient, scalable, cost-effective and sustainable model of training and consultation that can be transferred from experts to the school district is needed. A number of studies have shown that more experienced school personnel can successfully serve as coaches and supervisors to those implementing EBPs [25–27]. Sustainment of Tier 2 interventions is complicated by the fact that most urban schools do not have an established training approach for professionals already working in the schools [11, 17]. The lack of access to effective EBP training in low-income communities is contributing to major service disparities.

Based on the extant literature, our sustainment approach will (a) prepare for sustainment during initial implementation, (b) use existing supervision arrangements in the schools to develop and reinforce internal capacity for training and supervision, (c) train with the goal of maintaining high fidelity, (d) use implementation champions (i.e., district coaches already present at each school), and (e) maintain *buy-in* and support by instituting an Advisory Board of school district stakeholders.

We will test two strategies for sustainment: (a) *Prepare for Sustainment (PS)*—sustainment supported by school district coaches who, in turn, receive technological enhanced consultation from external consultants, and (b) *Sustainment as Usual (SAU)*—sustainment supported by school district coaches alone. The first strategy involves gradual removal of support coupled with the use of technology. This innovative strategy reduces cost of implementation while concurrently creating internal capacity for sustainment.

### Preliminary studies

The three EBPs that will be used in this study have been found to be effective in multiple studies. Also, our group has found the interventions to be effective specifically with students in the School District of Philadelphia (SDP) [7, 8, 28]. Our training and consultation approach has been used in two prior randomized trials in the SDP and shown to be feasible, clinically-effective and acceptable by therapists and supervisors [7, 8, 28]. We contacted administrators and school counselors from schools participating in a study using mental health EBPs embedded within PBIS to determine whether schools continued to implement the EBPs after the end of the study. We found that 50% of the schools continued to implement one of two EBPs one year after the end of the study, and 33% of schools continued to implement one of the EBPs two years after the end of the study. Only one school continued to implement both programs during both years. The main reason given for the discontinuation was lack of training support during the sustainment period.

In one of our prior studies, research staff provided interactive consultation to clinical supervisors on how to conduct effective supervision of school-based therapists on the implementation of two group therapy EBPs. The consultation for one of the conditions was conducted via Zoom. Therapists or research assistants video-recorded child sessions and supervision sessions. The videos were uploaded onto a dedicated server. A usability analysis of Zoom, plus the dedicated server and REDCap used in 12 schools, showed that Zoom was compatible with school and hospital computers as well as mobile devices. All scheduled consultation sessions were completed within the expected timeline and we were able to securely upload, view, and store all recorded session content on a dedicated server at the hospital. This technology allowed our team to successfully provide secure remote consultation, including video feedback and access to training and review materials, to agency supervisors beyond the initial on-site training workshop. We will use similar processes and technologies in the present study.

In our prior studies, we found that schools, coaches and Tier 2 implementers differ widely with regard to their exposure to EBPs, and their experience supervising other therapists on the implementation of mental health interventions. As such, members of the research team, who are highly experienced in the implementation of the EBPs used in the study and in training Masters-level therapists, will conduct the initial training and subsequent consultation in order to ensure that all schools and all supervisors and implementers are equally prepared for the sustainment phases of the study.

### Mediators and moderators of consultation type on fidelity

It is important to identify mechanisms (mediators) through which implementation strategies (i.e., consultation type) work and specific moderation factors that can be used to target implementation strategies to the appropriate Tier 2 implementer. In addition, it is critical to study the role of perceived appropriateness, feasibility and acceptability of consultation support and the individual level variables related to how Tier 2 implementers perceive their jobs because these constructs are modifiable. We are interested in the role of perceived consultation appropriateness (our main mediator variable) because this construct has been shown to be a “leading indicator” of implementation success [29]. We plan to examine whether a consultation strategy for sustainment implemented by school district coaches, who are in turn supported by expert trainers, would be more appropriate for Tier 2 school personnel and thus associated with higher EBP fidelity, than a consultation strategy implemented by school district coaches without support. We are also interested in assessing moderator effects of certain individual-level variables on EBP fidelity. Perceived burnout (emotional exhaustion, depersonalization, personal accomplishments), role conflict, and role overload have been shown to affect school personnel’s ability to teach and implement EBPs in the school setting [17, 30]. In our previous work, we have found that school personnel in under-resourced schools often feel overextended and exhausted and, in some cases, this affects their performance in implementing EBPs [8, 28]. Thus, we also will examine whether the relationship between perceived appropriateness and EBP fidelity varies depending on staff perceived burnout. Regardless of the specific results, by testing the hypothesized relationships, the findings will directly inform the design of future, targeted interventions. In particular, the findings will indicate whether interventions should focus on modifying the training and consultation approach in order to make them more appropriate, feasible and/or acceptable.

## Methods

### Participants

Participants will be school staff and students attending urban public schools that are already implementing PBIS in Philadelphia, PA.

### Inclusion criteria

The inclusion criteria for participants include:

#### Tier 2 team

School personnel already assigned by the school principal to the Tier 2 team to support students in Tier 2. The Tier 2 team will conduct team meetings to review referrals for Tier 2, determine appropriate interventions for students, and track student progress.

#### Tier 2 coach

School or district personnel with Masters-level education selected by administrators. The Tier 2 coaches will train and supervise Tier 2 team members and Tier 2 implementers during the sustainment phases.

#### Tier 2 implementer

These are school staff with and without prior mental health training selected by the school principal. Tier 2 implementers will implement Tier 2 interventions.

#### Tier 2 student

These students must be (a) attending one of the participating schools, (b) in grades 4–8; (c) identified by the Tier 2 Team as not responding to Tier 1 intervention, thus needing Tier 2 support; and (d) scoring > 1 standard deviation above the mean on the Emotional Symptoms or Conduct Problems of the Strengths and Difficulties Questionnaire (SDQ) [31] completed by a parent or a teacher. Students will receive Tier 2 support by participating in one of the Tier 2 interventions.

### Exclusion criteria

School personnel not employed by the school district and students not enrolled in Tier 2 will not participate. Students with a special education classification of “Intellectual Disability,” students who have diagnoses that make participation in the study clinically inappropriate (i.e., current substance abuse disorder, psychotic or autism spectrum disorders, based on school records) because they would be unlikely to benefit from the interventions used in the study or who present as an acute risk to themselves or others, will be excluded.

### Overview and randomization

**Fig. 1:**
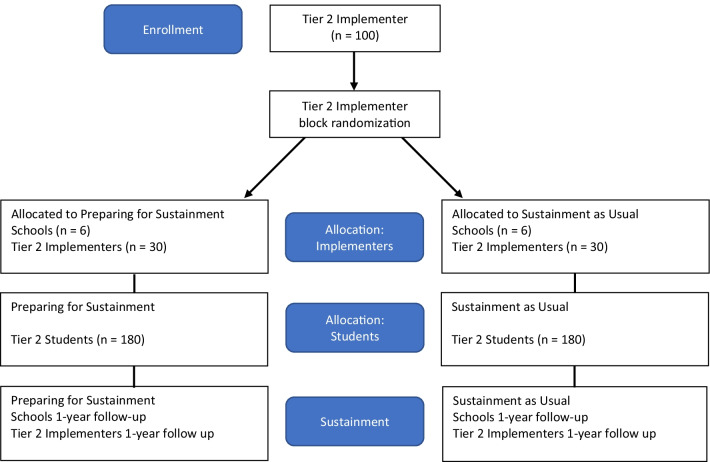
CONSORT diagram

The study uses a parallel 2-arm, cluster randomized controlled trial design. The two arms are: (a) Prepare for Sustainment (PS), and (b) Sustainment as Usual (SAU) (see Fig. 1).

We will recruit 12 schools, 145 school personnel (13 coaches, 72 Tier 2 members, 60 Tier 2 implementers), and 360 students in 3 waves (4 schools, 20 implementers, 120 students in each wave) in order to strategically deploy resources. During Phase 1 (all waves), Tier 2 implementers and Tier 2 team members will receive the same level of support provided by research consultants. At the end of Phase 1 of each wave, schools will be randomly assigned in 1:1 ratio to condition (PS [n = 6], SAU [n = 6]). Tier 2 implementers, school district coaches, and Tier 2 team members will participate in their school’s assigned condition. We expect that there will be 5 Tier 2 implementers per school. Schools will be stratified by school level (elementary or middle schools) to ensure that both conditions have the same number of school types. All schools will have similar levels of students qualifying for free or subsidized lunch.

### Tier 2 interventions

All participating schools will already be implementing PBIS at Tier 1. None of the schools will have any significant experience prior to implementing mental health EBPs at Tier 2. The EBPs that schools will be able to use for Tier 2 in this study are shown in Table 1.

**Table 1:** Tier 2 interventions

Intervention	Description	Number of sessions	Implementer
Coping Power Program (CPP) [32]	Group intervention for children with, or at risk for, externalizing behavior problems [33, 34]	12	Counselor
CBT for Anxiety Treatment in Schools (CATS) [35]	Culturally-appropriate adaptation of Friends for Life (FRIENDS) [36] and *Coping Cat* [37] for children with, or at risk for, anxiety disorder	8	Counselor
Check-in/Check-out (CICO) [38]	Targeted intervention for students at risk of developing externalizing and internalizing mental health disorders. [38, 39]	Variable, depending on need	Other school staff

Decisions as to whether students are assigned to group intervention or individual intervention will be made by the Tier 2 team in each school. These decisions will be largely based on whether the Tier 2 team can assemble intervention groups of students of similar developmental level who present similar difficulties. In addition, the Tier 2 team might choose individual or group therapy for certain students based on the student’s presenting problems or other factors important to the school. The study team will provide guidance to the Tier 2 team about the type of students who would be appropriate for each specific program or for individual vs. group intervention.

### Training components

**Fig. 2:**
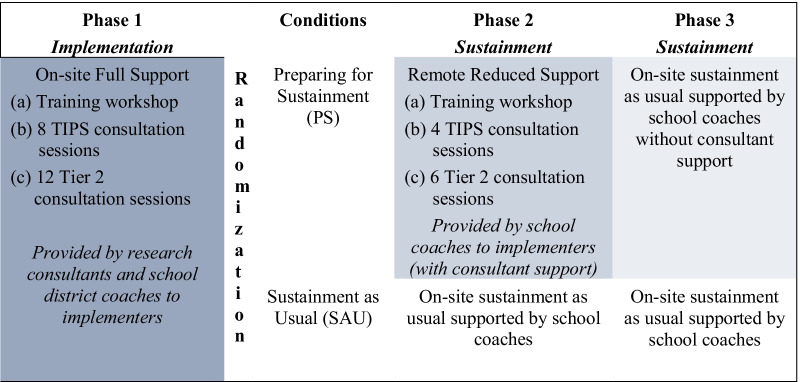
Sustainment Support for School Personnel

Initial training is provided by research team consultants. Initial training components for all schools (Phase 1) will include (a) initial training workshop, (b) consultation on the use of the Team-Initiated Problem Solving (TIPS) [40] approach for assigning students to a specific Tier 2 intervention, and monitoring students’ progress, and (c) consultation on the use of Tier 2 interventions (CPP, CATS, CI/CO). Subsequent training support (Phases 2–3) will vary by year and condition (see Fig. 2). All materials, including Tier 2 interventions, training manuals and exemplar videos, will be accessible via an online platform to all participating school personnel. The purpose of Phase 1 is to create a baseline where all participating school personnel will be equally prepared for the sustainment phases.

### Preparing for sustainment (PS) condition

Following Phase 1 (implementation), schools will be randomly assigned to PS or to SAU. Following the retraining workshop during Phase 2, school district coaches (with research consultant support) will provide 5 separate on-site consultation sessions to each Tier 2 team; and 6 on-site sessions for Tier 2 implementers. The consultation sessions will be pre-scheduled at the beginning of the school year. With regard to content, the Tier 2 team will be expected to choose a particular Tier 2 team audio-recorded meeting and an intervention session that the team would like the school district coach to review and discuss during the consultation session. Also, the Tier 2 implementers will be expected to raise points that they would like to discuss and receive feedback regarding any aspect of the intervention (e.g., how to conduct a particular role play in session) or any implementation barrier. The remote support will be provided to the school district coaches via Webex and an online file share server will include consultation manuals, materials (e.g., TIPS forms for running effective Tier 2 team meetings using the TIPS approach; fidelity forms for Tier 2 interventions), and how to prepare for the consultation session with school personnel. The same coaches in PS will support the schools during Phase 3 without receiving any support from external consultants.

### Sustainment as usual (SAU) condition

During Phases 2–3 of the study, school district coaches will provide separate on-site consultation to the Tier 2 team and Tier 2 implementers using a sustainment as usual approach. School district coaches will have participated in a training for coaches and as active participants during the initial training and consultation meetings for Tier 2 teams and Tier 2 interventions (Phase 1) for school personnel in SAU. They will not receive direct support from external consultants but they will have access to all training and consultation materials.

#### Training of coaches

Across the three study waves, 6 Tier 2 coaches (3 coaches in PS and 3 coaches in SAU) will participate in a 2-day training workshop on supervision strategies for the implementation of EBPs in school settings (e.g., learn a competency framework for supervisors [41], strategies for identifying children who could benefit from the service, and how to track their progress). They will also be trained on conducting consultation with Tier 2 implementers. External consultants from the research team will provide 1 h of consultation support to school district coaches in the PS condition for each consultation session school district coaches provide to school personnel during Phase 2. The district coaches will also assist research consultants during Phase 1 as they train and provide consultation to Tier 2 implementers. The 6 district coaches will support Tier 2 implementers during Phase 2 and Phase 3.

### Implementation framework and training procedures

**Fig. 3:**
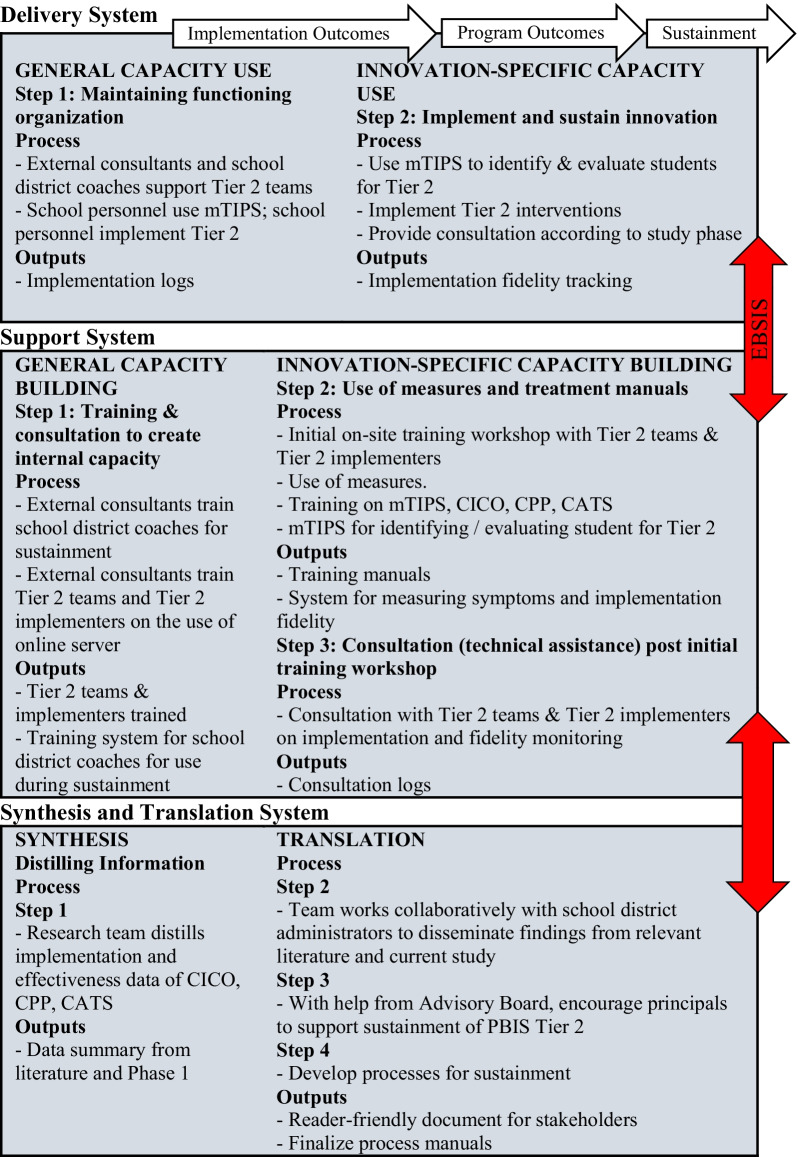
Interactive Systems Framework for D&I

All activities related to the training of school personnel and implementation and sustainment of EBPs are guided by the Interactive System Framework for Dissemination & Implementation (ISF) [42] (see Fig. 3). ISF is intended to be a “heuristic for understanding key systems, key functions, and key relationships relevant to the dissemination and implementation process” [42] (p. 179). ISF is composed of three interrelated systems: Synthesis and Translation System (STS), Support System (SS), and Delivery System (DS). The function of STS is to distill information innovations and prepare them for implementation by service providers. SS supports the work of those who put the innovation into practice. The primary function of DS is the implementation of innovations in “real world” settings [42, 43].

#### Synthesis and translation system (STS)

An Advisory Board will be created to facilitate recruitment and buy-in, and to employ “climate embedding mechanisms” [44] for the implementation and sustainment of EBPs in the school district. The Advisory Board will be comprised of school district administrators, principals and counselors.

#### Synthesis (step 1) & translation (step 2)

We will present synthesized information about the implementation and effectiveness data of Tier 2 EBPs from literature reviews and previous studies conducted by our research team to the Advisory Board. We will work with the Advisory Board to create an easy-to-read document that shows the effectiveness of Tier 2 EBPs used in the study and share it with school staff and parents in order to seek stakeholder buy-in for sustainment. *(Step 3)* We will seek principal commitment to support and champion the integration of mental health supports at Tier 2. Based on our preliminary studies and PBIS literature, we will employ the TIPS approach to support the school Tier 2 teams, which will include Tier 2 implementers, as they incorporate Tier 2 mental health supports. *(Step 4)* We have developed training materials that will be disseminated among participating schools. The training materials include a series of video modules. Modules that focus on interventions include a discussion of the theoretical background of the particular EBP, its development and a detailed review of group sessions. Participants in both conditions will have access to these materials for the duration of the project via a dedicated online server housed at CHOP.

### Support system (SS)

#### General capacity building (step 1)

The study will be conducted in the context of general capacity building for sustainment, specifically, training and consultation, to enable certain school personnel (e.g., faculty, counselors, social workers, climate specialists) to become effective users of the TIPS process for identifying and following students in Tier 2. Activities in the Support System and the bidirectional relationship of activities between the Support System and the Delivery System will be informed by the Evidence-Based System for Innovation Support (EBSIS) [43], a logic model developed by Abraham Wandersman and colleagues, to supplement the ISF [42, 43]. The EBSIS consists of a series of four sequential support components: *Training, Tools, Technical Assistance, and Quality Assurance/Quality Improvement* [43].

#### Training

Research consultants will be project staff with expertise in the implementation of PBIS with mental health supports. They will be trained and supervised by senior members of the research team on how to conduct training, retraining and consultation. Research consultants will conduct three days of on-site training in August at the outset of the study (Phase 1) for all school personnel involved in the TIPS approach and Tier 2 EBPs. District coaches will conduct retraining for PS after having received consultation from external consultants (Phase 2). District coaches in SAU will conduct retraining by themselves. Members of the Tier 2 teams and Tier 2 implementers will be introduced to the TIPS [40] approach to use data to identify and assign students at risk for behavioral and emotional disorders into Tier 2, and to measure fidelity to the EBP and intervention outcomes. The Tier 2 implementers will also be taught a competency framework [41, 45], strategies (e.g., in-service presentations to the faculty) for enhancing school personnel knowledge of mental health “red flags” among students, and how to access the online materials. Retraining (Phases 2–3) will include a refresher of the information contained in the initial training.

#### Innovation-specific capacity building, (step 2)—tools

During the initial training (Phase 1) and retraining, members of the Tier 2 team/Tier 2 implementers and district coaches will be instructed to use training manuals and adherence checklists for TIPS and Tier 2. The Tier 2 team will be trained on the use of a mental health screening instrument (SDQ) [31] and a parent rating scale (Behavioral Assessment System for Children, Third Edition [BASC-3]) [46] and other instruments used in Tier 2. Based on our prior studies and informed by TIPS [40], the Tier 2 team will also learn how to modify the standard PBIS leadership team meetings to integrate mental health interventions into the tiers of support, context for problem behaviors, how to identify students in need of higher levels of support, CICO, and other components used in PBIS training. [45, 47] During all phases of the study, members of the Tier 2 team will be expected to include Tier 2 implementers in their meetings and to support them by discussing, and solving as a group, any Tier 2 implementation barriers. Tier 2 implementers will be introduced to a competency model for CBT [48]. They will also learn about how to deal with implementation barriers (e.g., scheduling sessions, conducting exposure tasks) [49].

### Delivery system (DS)

#### General capacity use (step 1)—technical assistance

During Phase 2 and Phase 3, Tier 2 teams and Tier 2 implementers, respectively, will receive a 3-h retraining by school district coaches. The purpose of the retraining will be to review key points from the training and to answer questions about any aspect of the implementation process. Through the initial training and retraining, the Tier 2 teams will have the capacity to use the TIPS process [40] to identify students using SDQ data. Tier 2 implementers will have the capacity to implement CI/CO and group CBT with fidelity.

#### Innovation-specific capacity use, (step 2)—consultation

Training and consultation content and procedures will be based on adult learning characteristics (e.g., propensity to learn from experience, capacity to reflect on performance and apply knowledge, and self-motivation) [50, 51]. Consultants will provide eight 50-min training sessions on assisting the Tier 2 team to run team meetings using the TIPS process. Consultation for Tier 2 will be provided for each intervention the Tier 2 implementer conducts. For example, consultants will provide one 50-min consultation session to Tier 2 implementers for each group session the staff member conducts with students (e.g., 12 sessions for Coping Power Program). Consultation for TIPS will include: (a) reflecting [52] on a prior team meeting using TIPS; (b) providing feedback based on TIPS fidelity measure; and (c) dealing with implementation barriers. Consultation for Tier 2 will include: (a) reflecting on previous group intervention session or CI/CO case; (b) discussion of intervention strategies; and (c) tailored problem solving for dealing with implementation barriers. In addition, consultation will dedicate time for didactics (e.g., preparing for next team meeting or group CBT session or CI/CO case; answering questions about the consultation manual).

#### Quality assurance / quality improvement

All Tier 2 team meetings and student Tier 2 group sessions will be audio-recorded using a device provided by the research team. A designated member of the Tier 2 team and Tier 2 implementers will be in charge of audio-recording and uploading audio files to an online server. If schools have difficulty doing this, the audio-recording and uploading will be conducted by a research assistant (RA). The audio files will be coded by independent coders (ICs) for fidelity. About 20% of the audio files will also be listened to by external consultants during Implementation; school district coaches in PS will listen to the audio files for the purpose of consultation. These procedures will ensure quality assurance / quality improvement for the consultation approach used in the study and should contribute to maintaining appropriate implementation and clinical outcomes. The audio files will be provided to school district coaches in SAU and to all district coaches during Phase 3 so that they can continue to be used to provide feedback to the Tier 2 team and Tier 2 implementers. Given that district coaches will operate totally independently of the research team in SAU and Phase 3 of PS and SAU, the research team will keep track of the use of audio files in consultation, as well as the frequency and length of consultation sessions via logs completed by school district coaches.

### Measures and assessment procedures

Thought leaders and researchers in the area of sustainment [53] have identified four characteristics of optimal sustainability research: (1) using mixed quantitative and qualitative data approaches; (2) reflecting perspectives of multiple stakeholders invested; (3) capturing variables at multiple levels of health delivery; and (4) collected over multiple time points. We will use this approach to data collection. A list of measures is provided in Table 2.

**Table 2:** Measures by variable/construct, measure characteristics, timepoint, method and informant

Variable/construct	Measure	Measure characteristics	Timepoint	Method	Informant
**Pre-trial activities**					
Fidelity to TIPS	Modified Team Initiated Problem Solving Checklist (mTIPS) [54]	The mTIPS has 9 meeting foundation items, and 6 problem solving items, scored on a three-point scale (e.g., 0 = No problem is defined; 1 = At least one problem is defined but lacks one or more precision elements; 2 = At least one problem is defined with all precision elements)	Variable	Coding	Research staff
Tier 2 screening	Strengths and Difficulties Questionnaire (SDQ) [31] with Impact Supplement [55]	The SDQ is a 25-item, 3-point scale (0 = not true; 2 = certainly true) questionnaire used to assess the psychological adjustment of children and youth, ages 4–17	Pre-treatment	Rating scale	Parents/teachers
**Adherence to training strategies**					
Consultation adherence	Internal Consultation Adherence Measure (ICAM)	The ICAM is an 8-item rating scale measuring adherence to Coaching (e.g., The Internal Trainer encouraged the counselor to deliver content using active learning strategies such as role play) and Didactics (e.g., The internal Trainer reviewed the specifics of the activities of the upcoming session and helped counselor prepare) rated on a 4-point scale (1 = minimal, 2 = some, 3 = substantial, 4 = N/A	Ongoing	Quan	Research staff
Tier 2 Team coaching fidelity	Tier 2 Coaching Fidelity Checklist (Tier 2 CFC)	The Tier 2 CFC is a 7-item checklist assessing coaching fidelity (e.g., Coach attends meeting on time and observes entire Tier 2 team meeting) rated on a yes/no scale	Ongoing	Quan	Research staff
**Aim 1: Compare sustainment conditions** **Implementation measures**					
Content fidelity of group CBT	Coping Power and CATS Content Fidelity Checklist (CFC) [7]	The CFC reflects each activity component of the session agenda of the treatment protocols. Raters use a yes/no response scale to indicate whether or not the implementer covered a particular component	Ongoing	Coding	Research staff
Content fidelity of CI/CO	Check-In/Check-Out Fidelity Checklist [56]	The Check-In/Check-Out Fidelity Checklist is a 9–10-item checklist used to observe the Tier 2 implementer during morning check-in and afternoon check-out rated as either occurring or not occurring	Weekly	Coding	Research staff
Process fidelity of Tier 2	Process Fidelity Checklist (PFC) [7]	The PFC is a 10-item checklist rated on a scale of 1 to 5, with 1 being *Not at all* and 5 being *Very Often*. Ratings are given on the extent to which school staff members delivered the intervention in an orderly fashion, using active learning strategies and examples that are relevant to the students	Weekly	Coding	Research staff
Fidelity to Tier 2 structures and processes	Tiered Fidelity Inventory (TFI) for Tier 2 [57]	The Tier 2 section of the TFI has 13 items organized into three subscales: (a) teams, (b) interventions, and (c) evaluation. School teams use a 3-point scale with supporting data sources and a detailed rubric to determine whether the core feature addressed in each item is *not implemented*, *partially implemented*, or *fully implemented*	Monthly	Coding	Tier 2 team
Penetration	Penetration Questionnaire (PQ)	The PQ is a 2-item questionnaire assessing EBP penetration at the school provider level (behavioral health staff involved in the implementation of EBPs at Tier 2 of PBIS), and EBP penetration at the student service level (students receiving EBPs at Tier 2)	Monthly	Quan	Tier 2 coaches
**Student outcome measures**					
Mental health symptoms	Behavior Assessment System for Children- 3^rd^ Edition (BASC-3) [46]	The BASC-3 is a 138-item, 4-point, Likert-type rating scale for assessing parental report of child mental health functioning, standardized for ages 2.5 to 18 years	Pre/Post-treatment	Rating scale	Parent
	The Behavior and Feeling Survey-Youth Report (BFS-YR) [58]	The YFS-SR is a 10-item measure of internalizing and externalizing problems rated on a 5-point scale (0 = not a problem; 4 = a very big problem)	Pre/Post-treatment	Rating scale	Child
Student academic engagement	Engagement versus Disaffection with Learning- Child Report (EvsD-Teacher) [59, 60]	The EvsD Child is a 20-item, four-point (1 = not at all true; 4 = very true) instrument with four sub-scales: Behavioral Engagement, Emotional Engagement, Behavioral Disaffection and Emotional Disaffection	Pre/Post-treatment	Rating scale	Child
	Engagement versus Disaffection with Learning- Teacher Report (EvsD-Child) [59, 60]	The EvsD Teacher is a 20-item, four-point (1 = not at all true; 4 = very true) instrument with four sub-scales: Behavioral Engagement, Emotional Engagement, Behavioral Disaffection and Emotional Disaffection	Pre/Post-treatment	Rating scale	Teacher
**Cost**					
Implementation cost	REDCap time tracking	The time tracking tool captures training time for school personnel and school district coaches, as well as for those providing external support (external consultants); time subsequently spent in consultation by school district coaches; time spent by outside external consultants consulting with school district coaches; and time implementing Tier 2 interventions; as well as assessment and documentation time	Ongoing	Survey	School staff
	DATCAP interview [61]	School staff are interviewed to capture other program costs	Post	Interview	School staff
**Aim 2: Mediators and Moderators**					
Mediation	Acceptability of Intervention Measure [AIM] [62]	The AIM is a 4-item instrument rated on a 5-point Likert type scale (1 = completely disagree, 2 = disagree, 3 = neither agree nor disagree, 4 = agree, 5 = completely agree)	3 times per year	Rating scale	School staff
	Intervention Appropriateness Measure [IAM] [62]	The IAM is a 4-item instrument rated on a 5-point Likert type scale (1 = completely disagree, 2 = disagree, 3 = neither agree nor disagree, 4 = agree, 5 = completely agree)	3 times per year	Rating scale	School staff
	Feasibility of Intervention Measure [FIM) [62]	The FIM is a 4-item instrument rated on a 5-point Likert type scale (1 = completely disagree, 2 = disagree, 3 = neither agree nor disagree, 4 = agree, 5 = completely agree)	3 times per year	Rating scale	School staff
Moderation	Maslach Burnout Inventory Education Survey (MBI-ES) [63]	The MBI-ES is comprised of 22 items organized in three scales: Emotional Exhaustion (EE), Depersonalization in the Workplace (DW), and Personal Accomplishment (PA)	3 times per year	Rating scale	School staff
	Teacher Stress Scale (TSS) [64]	Role conflict and role overload will be measured using subscales of the TSS. The subscales are comprised of 5 items each rated on a 6-point Likert scale (1 = strongly disagree, 2 = disagree, 3 = not sure, 3 = agree, and 4 = strongly agree)	3 times per year	Rating scale	School staff
**Aim 3: Perceptions of Training Support**					
Perceptions of training support	Qualitative Interview Guides	Semi-structured qualitative interviews are conducted with Tier 2 implementers, school district coaches, and school and Tier 2 team leaders to elicit views and perspectives about the feasibility and acceptability of the sustainment strategies	Yearly	Interview	School staff

### Data analysis by aim and hypothesis

#### Aim 1

To compare sustainment conditions for Tier 2 mental health interventions.

We hypothesize that randomly assigned Tier 2 implementers to the PS condition will maintain higher implementation fidelity at Phase 2 and Phase 3 compared to implementers assigned to the SAU condition.

To address implementation fidelity in Aim 1, Hypothesis 1, the unit of analysis is the 60 implementers who are nested within schools; strategies for sustainment groups are PS and SAU and time is the repeated measure collected by the end of Phase 1 (before randomization), Phase 2, and Phase 3. The linear mixed-effects model will be utilized with main effects of strategies for sustainment (PS, SAU), time (Baseline, Phase 2, Phase 3) and the group by time interaction will be the independent variables predicting implementation fidelity. Implementers across each school and each intervention’s year will be pooled and the models will include an implementer-level random effect to account for variability among implementers. For each outcome measure, the mixed effects modeling approach for a 2-level mixed effects linear regression model will be presented below for illustration purposes using the notation and descriptions given by Donner and Klar [65]: *Y*_*ijl*_ = *U* + *G*_*i*_ + *V*_*ij*_ + *e*_*ijl*_; Where *i* = 1, 2 (PS, SAU); *j* = 1, 2, *…, ki (*School *j* in group *i)* and *l* = 1,2, *…, m*_*ij*_ therapist in group *I* school *j.* Therefore*, Y*_*ijl*_ represents outcomes for participant *l in school j condition i*. *U* = response grand mean; *G*_*i*_ = fixed effect of the intervention condition*; V*_*ij*_ = the random cluster effects which assumed to be normally distributed ~ N(0, σ^2^A); e_ijl_ is the error term assumed to be normally distributed ~ N (0, σ^2^_w_). The equation represents a mixed effects model since the strategies for sustainment occurs at the school level (the clusters = *j*) and participants are nested within schools. The intervention effect is fixed, and the cluster effect is random. The effects of *V*_*ij*_* and e*_*ijl*_ are assumed to be independent with a common intra-cluster correlation. The 2-level model presented above will be expanded to include students within implementers (level 3) as a cluster and its random effect will be estimated, assuming it is normally distributed ~ *N* (0, σ^2^*z*). In all models, we will consider adding pertinent covariates related to student level factors if randomization at the school level did not ensure comparability among implementors and/or students characteristics between the two groups.

#### Hypothesis 2

Compared to SAU, PS will maintain higher penetration at the school provider level (i.e., behavioral health staff involved in the implementation of EBPs) at Tier 2, and EBP penetration at the student service level (i.e., students receiving EBPs at Tier 2) during Phase 2 and Phase 3 of the study compared to Phase 1.

The information regarding penetration (Aim 1, Hypothesis 2) will be collected using questionnaires completed by school therapists and their coaches. The data will be presented descriptively, using mean, standard deviation, quartiles, minimum and maximum by PS and SAU. Changes in score between Phase 1 and Phase 2 and Phase 1 and Phase 3 will be presented using the 95% confidence intervals.

#### Hypotheses 3 & 4

At Phase 2 and Phase 3, students receiving mental health interventions in PS will show better symptom severity improvement and better academic engagement improvement compared to students receiving mental health interventions in SAU.

Regarding Aim 1, Hypotheses 3 & 4, we will determine the difference in student symptom severity improvement and academic engagement improvement between PS and SAU. Statistical analysis related to student outcomes will be done by phases. Phase 1 will be analyzed as a one group using pre/post changes in BASC-3 and descriptive statistics for EvsD. Phase 2 and Phase 3 will be analyzed using the mixed effect modeling approach described above with group, time (pre/post) and interaction term (group X time).

Differences in BASC 3 and EvsD between students receiving support by implementers under PS vs SAU will be estimated by mean ± standard deviation and the 95% confidence intervals (CI) of the differences and presented by Phase 2 and Phase 3.

### Power analyses

We used PASS 3 Power Analysis and Sample Size Software [66] to determine the power and minimum detectable implementation fidelity effect sizes (MDES) based on a sample of 12 schools with 5 implementers per school (60 implementers). The effect size refers to the differences in implementation fidelity between PS and SAU (ES = (|µ_1_ − µ_2_|/σ)) in Phase 2 and Phase 3. Using the empirical results from our prior study on the effects of PBIS with mental health supports [7], the intra-cluster correlation coefficient (ICC) was set to 0.05. A group sample of 6 clusters (schools) per PS and SAU with 5 implementers per cluster, (i.e., 30 implementers in PS and 30 implementers in SAU; 60 total) achieve 85% power to reject the null hypothesis of equal implementation fidelity means when the population mean difference between PS and SAU is assumed to be 9.0%, standard deviations of 9.5, and with a significance level (alpha) of 0.05 using a two-sided two-sample t-test. We anticipate at total of 360 students will participate from 12 schools over three study phases (30 students per school).

#### Hypothesis 4

PS will be more cost-effective compared to SAU during Phases 2–3.

We will address Aim 1, Hypothesis 4 by calculating PS incremental cost-effectiveness ratio and determining whether it is significantly less than $150/[BASC-3 T-score]. This ratio is made up of the difference in cost divided by the difference in parent reported scores on the Aggression, Conduct Problems, and Anxiety scales of the BASC-3.

The difference in cost between PS and SAU will be assessed by use of the generalized linear model (GLM). Link and family functions for the GLM will be empirically fit to the data using diagnostic tests including the Modified Parks test, Pregibon-Link test, Hosmer–Lemeshow test [67]. Separate ratios will be calculated for Phase 2 and Phase 3 of the trial.

#### Aim 2

To explore mediators and moderators of consultation support on implementer fidelity.

**Fig. 4:**
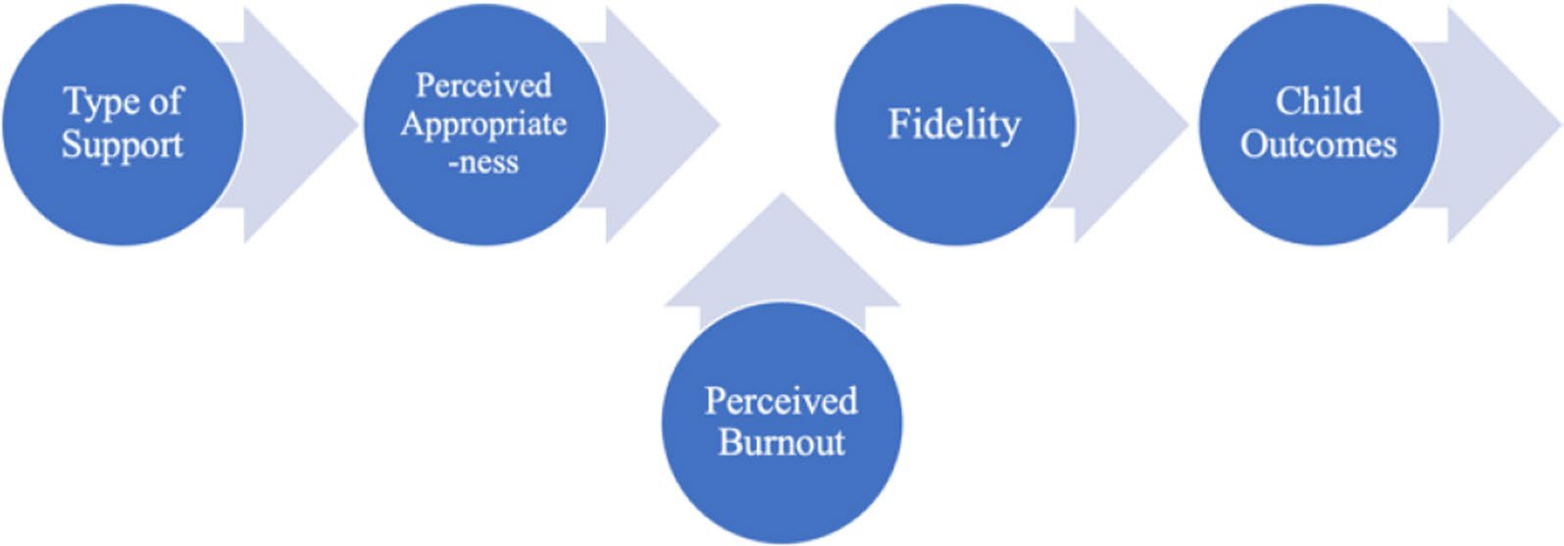
Mediators and moderators of type of support on fidelity

Our primary hypotheses are that (2a) Tier 2 implementers’ perceived appropriateness of the consultation support will mediate the relationship between type of support they receive and implementation fidelity, and (2b) implementer burnout will moderate the relationship between the type of consultation support and fidelity (see Fig. 4).

In addressing Aim 2, we will employ the regression modeling approaches proposed by Hayes [68]. These approaches utilize PROCESS macro suitable for SPSS and SAS. The models will reflect change scores in the mediators. To produce a precise estimate of mediation effects and properly account for the potential of regression to the mean, baseline measurement of the mediator will be included as a covariate.

To address the moderation effect, we will examine the impact of sustainment (PS vs. SAU) on implementation fidelity with the effect of implementer burnout on implementation fidelity. We will use a regression modeling approach with the outcome (Y) being the dependent variable, treatment condition (T) being the independent variable, a subgrouping variable or covariate (X) being the moderator tested, and the test of moderation reflected as the interaction of treatment by the covariate. The generic regression equation is Y = b_0_ + b_1_ T + b_2_ X + b_3_ (T*X). Where Y = implementation fidelity; T = sustainment group (PS, SAU), X = implementer burnout. A significant interaction term (T*X) of group by implementer burnout indicates that the treatment effect varies as a function of the covariate or subgroup variable. The Wald statistical test will be used for testing the interaction term. In planning and performing the analyses related to mediation and moderation, we will utilize the PROCESS modeling tool.

#### Aim 3

To qualitatively examine school personnel perceptions of mental health training support. Participants in Aim 3 will include Tier 2 implementers (n = 60), school district coaches (n = 6), and school leaders and administrators (n = 12). Interviews will be digitally recorded and transcribed with analyses supported by use of an NVivo database. Using an integrated approach [69] to codebook development, a priori codes will be developed to capture relevant implementation constructs including acceptability and feasibility of training and sustainment supports and implementation and sustainment processes. Additional codes will be added by the research team following a close reading of the first five transcripts [70]. We will develop a structured codebook. Each code will be defined and decision rules for their application included in the definition. Using NVivo, two members of the research team will separately code a sample of five transcripts and compare their application of the coding scheme to assess the reliability and robustness of the coding scheme. Disagreements in coding will be resolved through discussion and the codebook refined and applied to all transcripts. Coders will be expected to reach and maintain reliability at *κ* ≥ 0.85. Following coding, we will conduct member-checking, reviewing our conclusions with a multidisciplinary group of colleague clinical stakeholders to validate our analyses. Additionally, data will be triangulated with quantitative markers of feasibility, including proportion of schools and implementers invited to participate who enroll in the project and implementers retained throughout the sustainment phase.

#### Mixed methods analysis

We will integrate the quantitative data and interviews from Aim 1 using the following taxonomy: the structure is Quan → QUAL, the function is to expand upon the quantitative findings to understand the *process* of sustainment of Tier 2 interventions as experienced by stakeholders, and the process is connecting [71]. To integrate the quantitative and qualitative methods, we will follow the NIH guidelines for best practices [72]. We will use the quantitative data to identify patterns in the qualitative data. To do this, we will enter quantitative findings into NVivo as attributes of each participant (i.e., fidelity of the school the participant was drawn from). We will use the Content Fidelity Checklist (CFC) and the Check-In/Check-Out Fidelity Checklist as the measures of fidelity. The biostatistician will visually inspect the distribution of fidelity scores for each measure to determine logical cut points to classify schools as high, medium, and low fidelity and we will then enter these fidelity classifications into NVivo for each participant interview. These quantitative attributes will be used to categorize and compare important themes among subgroups.

## Discussion

We will compare the effects of two sustainment strategies on implementation and student outcomes. The examination of mediators and moderators of consultation support on fidelity might lead to the identification of modifiable factors to improve training and consultation approaches. We plan to use rigorous methods to compare outcomes, using measures with strong psychometric properties, multiple data collection strategies (surveys, interviews, independent coding), quantitative and qualitative data, and sound analytical methods.

### Innovations

This study contains several key innovations, including (a) examining sustainment, which is an understudied phase in implementation research in general, and in school mental health research in particular, (b) conducting the first study that seeks to test the effectiveness of a training and consultation support strategy for the sustainment of mental health EBPs within PBIS, (c) reducing cost for implementation while concurrently creating internal capacity for long-term sustainment, (d) using school stakeholders to prepare for sustainment concomitantly with initial implementation, (e) gradually removing supports in a way that is conducive to the transfer of knowledge from external consultants to school district coaches and Tier 2 implementers, (f) using the team problem-solving approach (TIPS) for mental health supports at Tier 2, and (g) conducting the first study investigating the cost and cost-effectiveness of two sustainment strategies in urban schools.

### Limitations

We will not be able to test the effectiveness of Tier 2 interventions against a control intervention. However, the Tier 2 interventions used in the study have been found to be effective in multiple studies. The current study will not be able to obtain implementation or effectiveness data on students needing individualized supports (Tier 3). Collecting these types of data would be beyond the scope of the current study. Results may not generalize to non-urban schools because of the unique characteristics of under-resourced urban schools. However, results should generalize to any large urban school district in the country.

## Conclusions

We expect that this study will result in a feasible, effective, and cost-effective strategy for the sustainment of mental health EBPs embedded in PBIS. Linking PBIS with state-of-the-art mental health supports and well-trained school personnel will likely lead to sustained improvement in student mental health and academic markers. Results from this study conducted in a large urban school district would likely generalize to other large, urban districts, and impact population-level child mental health.

## Data Availability

The datasets generated and/or analyzed during the current study will be available in the NIMH Data Archive repository, https://nda.nih.gov/.
